# MicroRNA-1-3p Suppresses Malignant Phenotypes of Ameloblastoma Through Down-Regulating Lysosomal Associated Membrane Protein 2-Mediated Autophagy

**DOI:** 10.3389/fmed.2021.670188

**Published:** 2021-05-26

**Authors:** Xing Niu, Biying Huang, Xue Qiao, Jinwen Liu, Lijie Chen, Ming Zhong

**Affiliations:** ^1^Department of Stomatology, Xiang'an Hospital of Xiamen University, Xiamen, China; ^2^Department of Oral Histopathology, Liaoning Province Key Laboratory of Oral Disease, School and Hospital of Stomatology, China Medical University, Shenyang, China; ^3^Department of Central Laboratory, Liaoning Province Key Laboratory of Oral Disease, School and Hospital of Stomatology, China Medical University, Shenyang, China

**Keywords:** ameloblastoma, miR-1-3p, Beclin1, LAMP2, LC3, autophagy, malignant phenotypes

## Abstract

**Objective:** Several clinical trials have suggested that autophagy inhibition is a promising approach for cancer therapy. However, the implications of autophagy in ameloblastoma (AB) remain undiscovered. This study investigated the dysregulated autophagy and its regulatory mechanisms in AB.

**Methods:** The expression and distribution of autophagy-related proteins including B-cell lymphoma-2-interacting protein-1 (Beclin1), microtubule-associated protein 1 light chain 3 (LC3) II/I and lysosomal associated membrane protein 2 (LAMP2) were detected in AB and normal oral mucosa (NOM) tissues by immunohistochemistry and western blot analyses. Under transmission electron microscopy, the autophagy of AB was observed. LAMP2 was a potential target mRNA of miR-1-3p. Quantitative Real-time PCR (qRT-PCR) analysis was utilized for examining LAMP2 and miR-1-3p in AB tissues as well as AM-1 cells. The correlation between LAMP2 and miR-1-3p was analyzed in AB. After transfection with miR-1-3p mimic or inhibitor, LAMP2 expression, proliferation, migration, and invasion were separately detected in AM-1 cells. Rescue assays were finally presented.

**Results:** Our results showed that Beclin1 was lowly expressed as well as LC3II/I and LAMP2 were highly expressed in AB. Autophagosomes were observed in AB. MiR-1-3p was lowly expressed in AB, which exhibited negative correlations to LAMP2 expression. MiR-1-3p up-regulation significantly lowered LAMP2 expression in AM-1 cells. Furthermore, miR-1-3p overexpression restrained proliferative, migrated, and invasive capacities of AM-1 cells, which were ameliorated by LAMP2 overexpression.

**Conclusion:** Our findings demonstrated that miR-1-3p suppressed malignant phenotypes of AB through down-regulating LAMP2-mediated autophagy, which could become an underlying target for AB therapy.

## Introduction

Ameloblastoma (AB), a common odontogenic epithelial neoplasm, exhibits locally invasive and aggressive behaviors (1). More than 80% of AB cases occur in the mandible (2). It has up to 90% risk of recurrence following conservative treatment (3). Although radical surgery can distinctly cut down the high recurrence risk, patients usually meet facial deformities (4). Unfortunately, the etiology of AB remains still unclear. Thus, it is of importance to research the molecular mechanisms of AB.

Autophagy is a stress response of eukaryotic cells to external pressure or stimulation such as starvation, hypoxia and toxicity (5). In the process of tumor development, autophagy may restrain the progression of tumors, but once the tumor is formed, autophagy may facilitate survival and growth of tumor cells (6). A previous study has found that LC-3 and p62 were highly expressed in primary AB-derived epithelial cells (7). Therefore, research on autophagy in odontogenic tumors is of great significance for understanding tumor occurrence and development. During autophagosome formation, microtubule-associated protein 1 light chain 3 (LC3) can be transformed from LC3-I to LC3-II, and LC3-II binds to the newly formed autophagosome membrane until the final autophagosome fuses with the lysosome (8). Therefore, LC3-II has become a marker of intracellular autophagy. Lysosomal associated membrane protein 2 (LAMP2) is a member of the membrane glycoprotein family, which protects the lysosomal membrane from hydrolysis by acidic hydrolases, and regulates membrane fusion of lysosomes with other organelles during autophagy (9). Additionally, B-cell lymphoma-2-interacting protein-1 (Beclin1) exerts a critical role in a critical step of the autophagic process. However, there are few studies on the expressions and clinical implications of autophagy-related proteins in AB.

MicroRNAs (miRNAs), with 21–22 nucleotides in length, may mediate gene silencing at post-transcriptional levels (10, 11). In various tumors, abnormal expression of miRNAs is involved in various biological processes (12). The ability to regulate miRNA expression and activity *in vivo* by its mimic or inhibitor offers a direction for developing innovative treatment strategies against cancers. MiRNA-based therapies may provide higher stability as well as protection from nucleases (12). Several miRNA-targeted therapies against cancers have entered clinical trials such as miR-34 (12). However, it is a challenge about how to select the optimal candidate miRNAs for each disease. Many studies have demonstrated that miRNAs can play regulatory roles in the processes of autophagy by mediating target genes (13–15). Among them, miR-1-3p is down-regulated in a variety of human tumors. For instance, overexpressed miR-1-3p restrains autophagy via targeting ATG3 in non-small cell lung cancer (16). Nevertheless, its expression and role in AB remain unclear.

Herein, our research investigated abnormally expressed autophagy-related proteins and analyzed their clinical implications. Our findings suggested that autophagy imbalance could be involved in the progression of AB. Furthermore, LAMP2 might become a potential target mRNA of miR-1-3p in AB.

## Materials and Methods

### Tumor Specimens

From 2014 to 2017, 12 paired AB and normal oral mucosa (NOM) fresh specimens were gathered from the oral and maxillofacial surgery of School and Hospital of Stomatology, China Medical University (Shenyang, China), which were then immediately stored at −80°C. Paraffin-embedded 104 AB tissues and 20 NOM tissues were retrieved from the Department of Oral Histopathology, School and Hospital of Stomatology, China Medical University between 2015 and 2016. This research strictly followed the Declaration of Helsinki. Each patient signed written informed consent. Our research gained the approval of the Ethics Committee of School and Hospital of Stomatology, China Medical University (2016-12).

### Immunohistochemistry

Paraffin-embedded tissue sections were cut to 100 μm thick, dried, deparaffinized and rehydrated following standard protocols. The sections were incubated with primary antibodies against Beclin1 (1:500; ab622557; Abcam, Cambridge, MA, USA), LC3 (1:2,000; ab51520; Abcam) and LAMP2 (1:1,000; ab25631; Abcam) at 4°C overnight. Then, the sections were incubated with secondary antibodies for 30 min at room temperature. The sections were stained by diaminobenzidine (DAB; Thermo Fisher Scientific, Waltham, MA, USA). Nuclei were lightly stained with hematoxylin. For negative control, the sections were treated as above but PBS (Hyclone, South Logan, UT, USA) instead of primary antibodies.

For each section, three fields were randomly selected (×200). The expression scores of Beclin1, LC3, and LAMP2 were on the grounds of staining intensity (no coloring: 0 point; light yellow: 1 point; brown yellow: 2 points; sepia: 3 points) and percentage of positive tumor cells (0–5%: 0 point; 6–25%: 1 point; 26–50%: 2 points; >50%: 3 points) (17). The final score was determined by staining intensity score × percentage of positive tumor cells (>4 scores: positive and 0–3 scores: negative).

### Western Blot

Tissues were lysed using 300 μl RIPA plus 3 μl protease inhibitor PMSF (Beyotime, Beijing, China) on the ice. After centrifugation for 5 min at 12,000 × g, the supernatant was stored at −20°C. The protein concentration was determined using a BCA protein assay kit (Beyotime). Total proteins in the supernatant were subjected to separation in 10% SDS-PAGE, followed by transference onto PVDF membranes (Millipore, USA). The membranes were blocked with 5% skim milk powder lasting 1 h at room temperature. Afterwards, the membrane was incubated by primary antibodies at 4°C overnight, and incubated with corresponding HRP-conjugated secondary antibodies (1:2,000; Abcam) for 50 min at room temperature in the dark. The primary antibodies included anti-Beclin1 (1:2,000; ab622557; Abcam), anti-LC3 (1:1,000; ab51520; Abcam), anti-LAMP2 (1:500; ab25631; Abcam) and anti-GAPDH (1:8,000; ab9485; Abcam). GAPDH was used as an internal control. The protein blot was visualized using Odyssey CLx.

### Quantitative Real-Time PCR

Total RNAs were extracted from tissues or cells with Trizol (Takara, Tokyo, Japan). For detection of miR-1-3p expression, cDNA was synthesized using TaqMan miRNA reverse transcription kit and specific primer of miR-1-3p. qRT-PCR was carried out on Hairpin-itTM Real-Time PCR Quantitation Kit. To detect the LAMP2 expression, cDNA synthesis was carried out using the SuperScript III. qRT-PCR quantification was performed using the SYBR Select Master Mix kit (Takara). β-actin and U6 served as internal controls of LAMP2 or miR-1-3p, respectively. The primer sequences of LAMP2, ATG3, β-actin, miR-1-3p, and U6 are shown in Table 1. LAMP2 and miR-1-3p expression was determined using 2^−Δ*ΔCT*^ method.

**Table 1:** The primer information for qRT-PCR.

**Target genes**	**Primer sequences (5'-3')**
LAMP2	5'-GTGAGTTGTATTGGGGTTGATGTTA-3' (forward)
	5'-CAATGATACTTGTCTGCTGGCTAC-3' (reverse)
β-actin	5'-TGGCACCCAGCACAATGAA-3' (forward)
	5'-CTAAGTCATAGTCCGCCTAGAAGCA-3' (reverse)
miR-1-3p	5'-CCACGATGGAATGTAAAGAAGT-3' (forward)
	5'-CAGAGCAGGGTCCGAGGTA-3' (reverse)
ATG3	5'-ACTGATGCTGGCGGTGAAGATG-3' (forward)
	5'-GTGCTCAACTGTTAAAGGCTGCC-3' (reverse)
U6	5'-ATTGGAACGATACAGAGAAGATT-3'(forward)
	5'-GGAACGCTTCACGAATTTG-3' (reverse)

### Transmission Electron Microscope

The tissues were washed using 0.1 cacodylate buffer (pH = 7.4), which were then fixed with PBS solution plus 3% glutaraldehyde as well as 2% paraformaldehyde. Standard protocols were presented for the rest of the process. The sections were stained with uranyl acetate and lead citrate. Images were captured under a transmission electron microscope.

### Cell Culture

Human immortalized AM-1 cell line that was gifted by Iwate Medical University (Japan) were cultured in Keratinocyte-SFM (Gibco, Invitrogen, USA). Human immortalized epidermal HaCat cell line that was purchased from Hunan Fenghui Biotechnology Co., Ltd. (Hunan, China) was cultured in DMEM (Hyclone, USA) containing 10% FBS at 37°C, 5% CO_2_.

### Transfection

MiR-1-3p mimic, inhibitor and corresponding miR-negative control (miR-NC) as well as LAMP2 and control pcDNA3.1 were purchased from GenePharma (Shanghai, China). The sequences were as follows: miR-1-3p mimics: 5'-UGGAAUGUAAAGAAGUAUGUAU-3'; miR-1-3p inhibitors: 5'-AUACAUACUUCUUUACAUUCCA-3'; miR-NC: 5'-UUCUCCGAACGUGUCACGUTT-3' or 5'-CAGUACUUUUGUGUAGUACAA-3'. AM-1 cells were seeded onto a 6-well plate for 24 h. Hundred nanometer mimic, inhibitor, or miR-NC as well as 3 μg plasmid were separately transfected into cells via Lipofectamine 2,000 reagent (Invitrogen, Carlsbad, CA, USA). Following transfection for 48 h, cells were harvested.

### Dual Luciferase Reporter Assay

The full or mutant fragments containing the predicted miR-1-3p binding sites in LAMP2 3'-UTR were cloned into the firefly luciferase gene in GP-miRGLO vector (Promega, Fitchburg, WI, USA). Afterwards, AM-1 cells were co-transfected miR-1-3p mimics or miR-NC with LAMP2 3'-UTR wild type (WT) or mutation (MUT). After 24 h, relative luciferase activity was determined through the Dual-Luciferase Reporter assay system (Promega).

### Cell Counting Kit-8

AM-1 cells were inoculated into a 96-well plate (1 ×10^3^ cells/well). After transfection with miR-1-3p mimic, miR-1-3p inhibitor and miR-NC or treatment with 3 mmol/L autophagy inhibitor 3-MA, cells were measured at 0, 24, 48, 72, and 96 h using CCK-8 kit (Dojindo, Kumamoto, Japan). The absorbance value at 450 nm was determined with a microplate reader.

### Colony Formation Assay

AM-1 cells were inoculated in a 10 cm dish (1 ×10^3^ cells/dish). Under culturing for 14 days, cells were fixed with methanol lasting 20 min, followed by staining with 0.1% crystal violet.

### Flow Cytometry for Apoptosis

AM-1 cells were inoculated onto a 6-well plate (4 ×10^4^ cells/well). Then, cells were centrifuged at 1,500 rpm for 5 min. Following washing with PBS for three times, cells were treated by 5 μL Annexin V-FITC as well as 5 μL PI lasting 10 min at room temperature away from light. Cell apoptosis was detected through flow cytometry.

### Transwell Assay

AM-1 cells (4 ×10^4^ cells/well) were inoculated onto the upper chamber in serum-free media with or without Matrigel. The lower chamber was added with culture medium. Under incubation lasting 24 h, migrated or invasive cells to the lower chamber were immobilized using 4% paraformaldehyde as well as stained using hematoxylin and eosin.

### Statistical Analyses

Statistical analyses were carried out utilizing Graphpad prism 7.0 and SPSS 17.0. Data are expressed as the mean ± standard error of mean (SEM). All assays were repeated three times. Comparisons between groups were presented with student's *t*-test or one-way analysis of variance. Chi square test was used for comparing the associations between clinical parameters and expression of proteins. The correlation between miR-1-3p and LAMP2 was evaluated via Spearman analysis. *P* < 0.05 was indicative of statistical significance.

## Results

### The Expression and Distribution of Beclin1, LC3, and LAMP2 in AB and NOM Tissues

**Figure 1:**
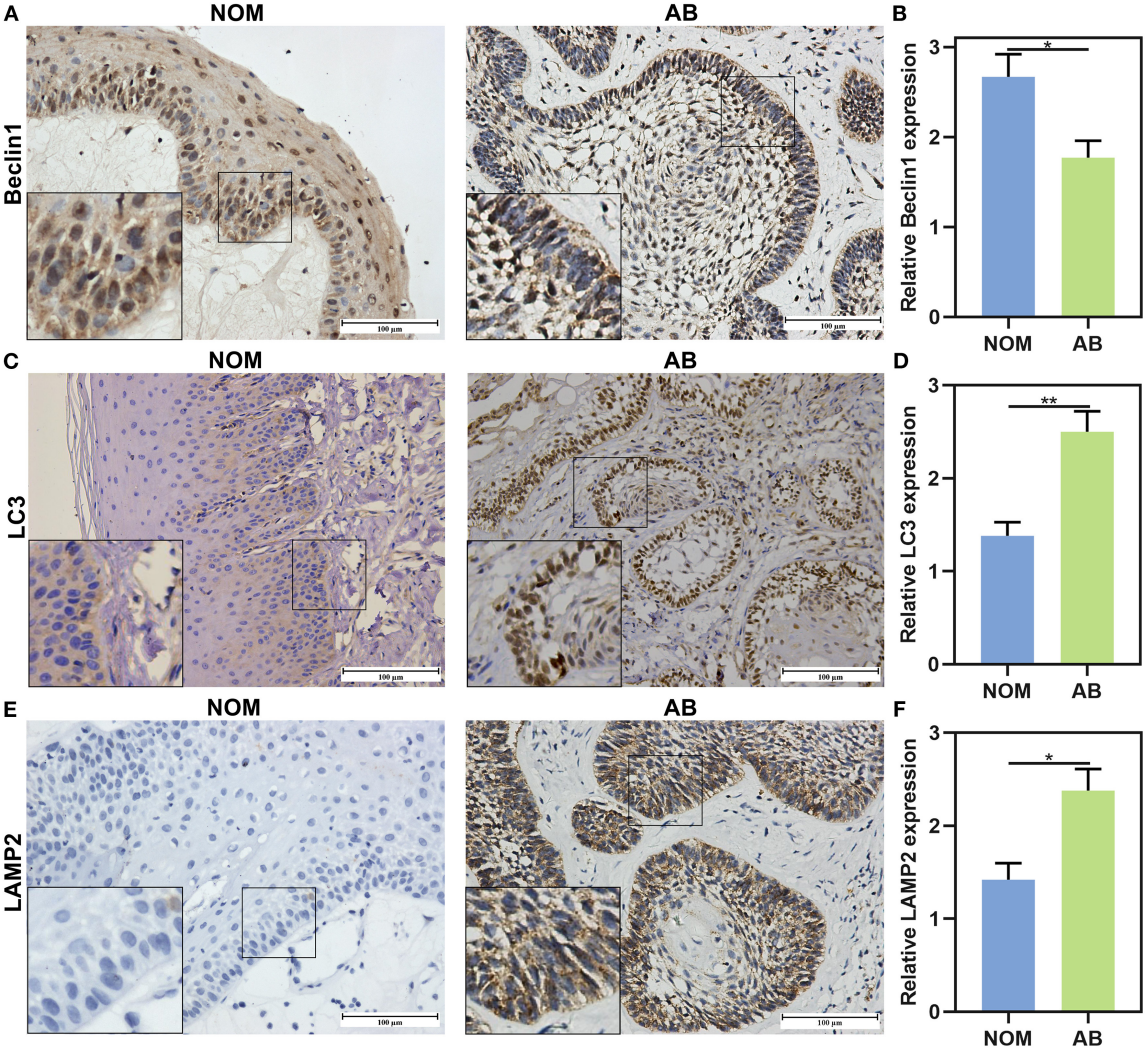
The expression and distribution of Beclin1, LC3, and LAMP2 in AB and NOM tissues by immunohistochemistry. **(A)** Beclin1 is positively expressed in the nucleus of epithelial cells in NOM tissues and is positively expressed in the cytoplasm of epithelial cells in AB tissues. **(B)** Quantitative analysis results showing a lower expression level of Beclin1 in AB tissues than in NOM tissues. **(C)** LC3 is negatively expressed in NOM and is positively expressed in the nuclei of epithelial cells in AB tissues. **(D)** Quantitative analysis showing a higher expression level of LC3 in AB tissues than in NOM tissues. **(E)** LAMP2 is negatively expressed in NOM and is positively expressed in the cytoplasm and cell membrane of epithelial cells in AB tissues. **(F)** Quantitative analysis for a higher expression level of LAMP2 in AB tissues than in NOM tissues. Bar: 100 μm. Magnification: 200 ×. *P < 0.05; **P < 0.01.

This study included 104 AB tissues and 20 NOM tissues. The expression and distribution of autophagy markers including Beclin1, LC3, and LAMP2 were assessed through immunohistochemistry. As shown in our results, Beclin1 was positively expressed in the nucleus of epithelial cells in NOM tissues (Figure 1A). Meanwhile, Beclin1 was positively expressed in the cytoplasm of epithelial cells in AB tissues. Quantitative analysis results confirmed that the expression of Beclin1 in NOM tissues was 1.51 times higher compared to that in AB tissues (*P* < 0.05; Figure 1B). The positive rate of Beclin1 expression in 104 cases of AB was 50% (52/104), which was significantly lower than that in NOM tissues (95%, 19/20). The correlations between Beclin1 expression and clinical pathological features were analyzed in AB. In Table 2, no significant difference was found between Beclin1 expression and age, gender, location, pathological classification, and recurrence of AB patients. In Figure 1C, LC3 was negatively expressed in NOM and the positive expression of LC3 was found in the nuclei of epithelial cells in AB tissues. LC3 expression in AB tissues was 1.82 times that of NOM tissues (*P* < 0.01; Figure 1D). The positive expression rate of LC3 in 104 cases of AB was 76.92% (80/104), which displayed a distinct higher level compared to that in NOM specimens (10%, 2/20). But no significant difference was detected between the expression of LC3 and age, gender, and recurrence of AB patients (Table 3). Intriguingly, we found that the positive expression rate of LC3 in the mandible of AB was significantly increased compared to that in the maxillary. Furthermore, the positive rate of LC3 in solid/multicystic AB exhibited a higher level in comparison to that in other types. In Figure 1E, LAMP2 was negatively expressed in NOM tissues while LAMP2 was primarily distributed in the cytoplasm and cell membrane of epithelial cells of AB. After quantification, LAMP2 expression in AB tissues was 1.68 times higher compared to that in NOM tissues (*P* < 0.05; Figure 1F). The positive expression rate of LAMP2 in 104 cases of AB was 63.46% (66/104), which was prominently more than that in NOM tissues (5%, 1/20; *P* < 0.05). In Table 4, LAMP2 expression in the mandible of AB was significantly higher than that in the maxillary, indicating that LAMP2 expression might be related to AB location. These findings indicated the dysregulated autophagy in AB.

**Table 2:** The association of Beclin1 expression with clinical pathological characteristics of AB.

**Clinical pathological characteristics**		**Cases**	**Beclin1 expression (*n*, %)**	**χ^2^**	***P***
Age	>50	31	17 (54.84)	0.414	0.520
	≤ 50	73	35 (47.95)		
Sex	Male	60	27 (45.00)	1.418	0.234
	Female	44	25 (56.82)		
Location	Mandible	84	43 (51.19)	0.248	0.619
	Maxillary	20	9 (45.00)		
Classification	Solid/multicystic	83	39 (46.99)	1.492	0.506
	Unicystic	12	13 (61.90)		
	Peripheral	6			
	Desmoplastic	3			
Recurrence	Yes	10	4 (40.00)	0.443	0.506
	No	94	48 (51.06)		

**Table 3:** The association of LC3 expression with clinical pathological characteristics of AB.

**Clinical pathological characteristics**		**Cases**	**LC3 expression (*n*, %)**	**χ^2^**	***P***
Age	>50	31	23 (74.19)	0.185	0.667
	≤ 50	73	57 (78.08)		
Sex	Male	60	46 (76.67)	0.005	0.942
	Female	44	34 (77.27)		
Location	Mandible	84	70 (83.33)	8.321	0.004^**^
	Maxillary	20	10 (50.00)		
Classification	Solid/multicystic	83	68 (81.93)	4.487	0.034^*^
	Unicystic	12	12 (57.14)		
	Peripheral	6			
	Desmoplastic	3			
Recurrence	Yes	10	8 (80.00)	<0.001	1.000
	No	94	72 (76.60)		

* *P < 0.05*;

** *P < 0.01*.

**Table 4:** The association of LAMP2 expression with clinical pathological characteristics of AB.

**Clinical pathological characteristics**		**Cases**	**LAMP2 expression (*n*, %)**	**χ^2^**	***P***
Age	>50	31	16 (51.61)	2.674	0.102
	≤ 50	73	50 (68.49)		
Sex	Male	60	36 (60.00)	0.733	0.392
	Female	44	30 (68.18)		
Location	Mandible	84	60 (71.43)	11.957	0.001^***^
	Maxillary	20	6 (30.00)		
Classification	Solid/multicystic	83	52 (62.65)	0.117	0.733
	Unicystic	12	14 (66.67)		
	Peripheral	6			
	Desmoplastic	3			
Recurrence	Yes	10	6 (80.00)	<0.001	1.000
	No	94	60 (63.83)		

*** *P < 0.001*.

### Dysregulation of Autophagy in AB Tissues

**Figure 2:**
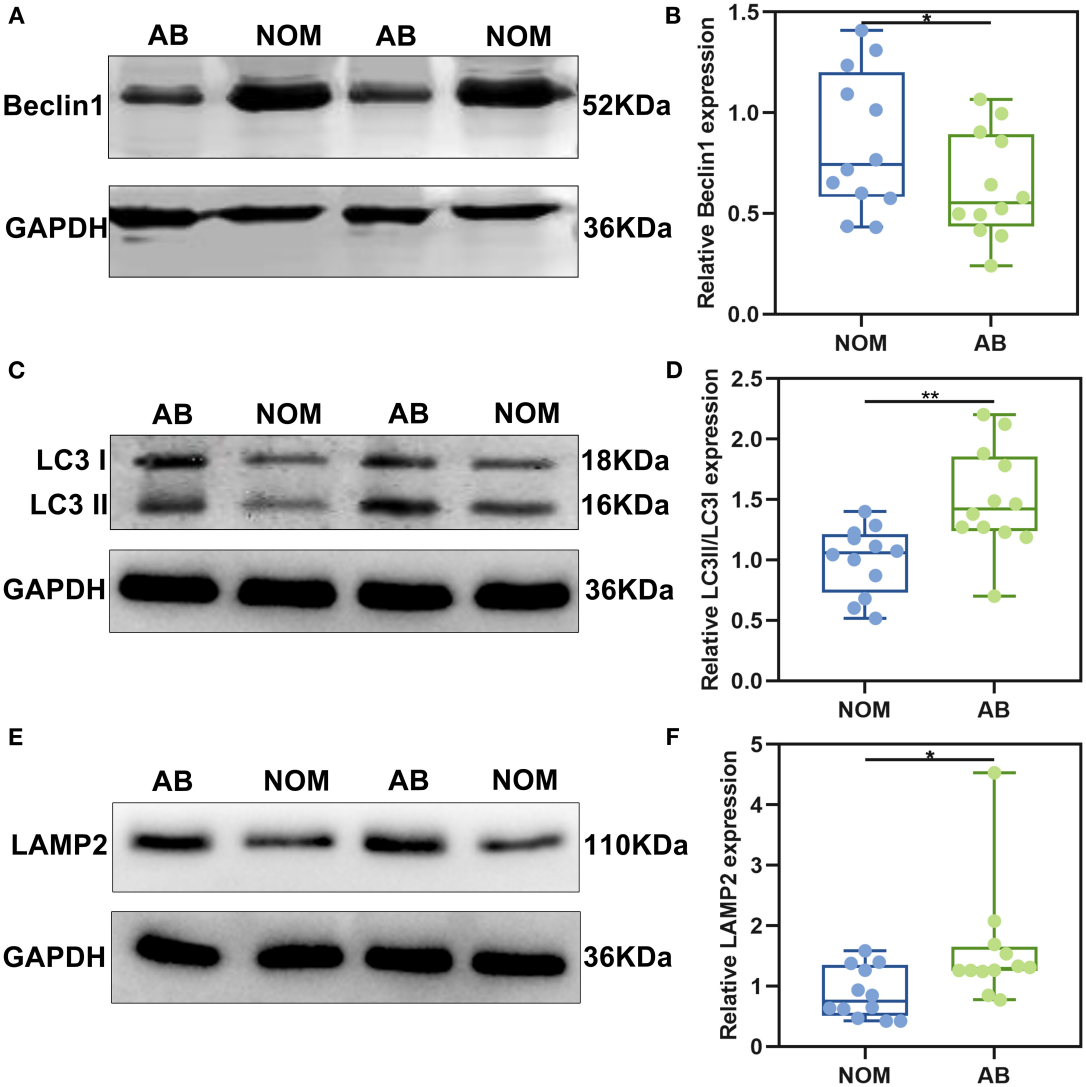
The expression of autophagy-related proteins Beclin1, LC3II/I, and LAMP2 in AB and NOM tissues by western blot. **(A,B)** Beclin1; **(C,D)** LC3II/I; **(E,F)** LAMP2. *P < 0.05; **P < 0.01.

**Figure 3:**
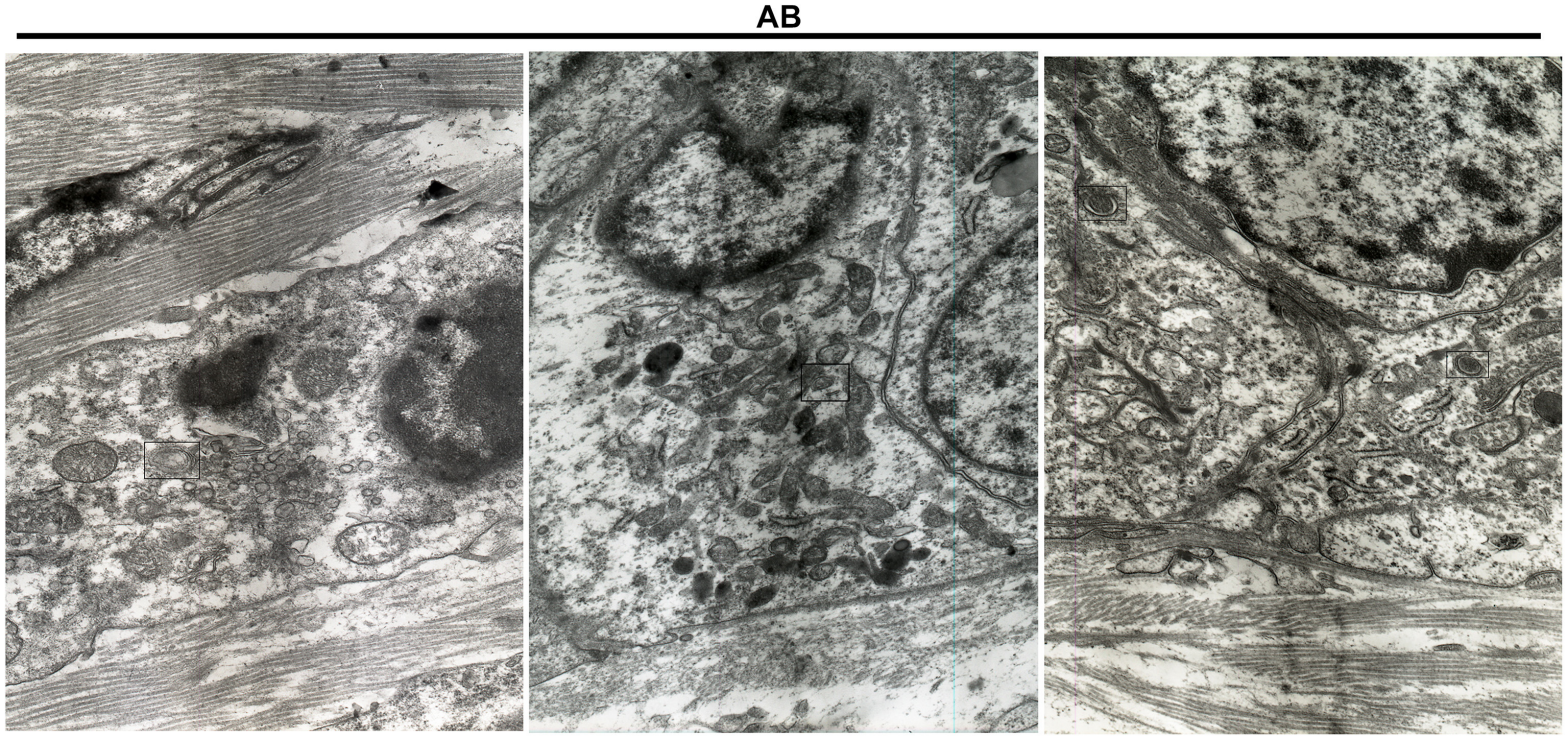
Transmission electron microscopy for the structure of autophagosomes in AB. The autophagosomes are marked.

After immunohistochemistry, we further the expression levels of autophagy-related proteins including Beclin1, LC3II/I and LAMP2 in AB and NOM tissues by western blot. Consistent with immunohistochemistry results, western blot analysis demonstrated that Beclin1 expression in AB tissues was distinctly lower compared to that in NOM specimens (*P* < 0.05; Figures 2A,B). Furthermore, LC3II/I in AB tissues was significantly higher in comparison to that in NOM specimens (*P* < 0.01; Figures 2C,D). As expected, overexpressed LAMP2 was detected in AB tissues in comparison to NOM specimens (*P* < 0.05; Figures 2E,F). Ultrastructure of autophagosomes was investigated in AB tissues under a transmission electron microscopy (Figure 3). Above findings demonstrated the autophagy activation in AB.

### LAMP2 Is a Target mRNA of miR-1-3p in AB

**Figure 4:**
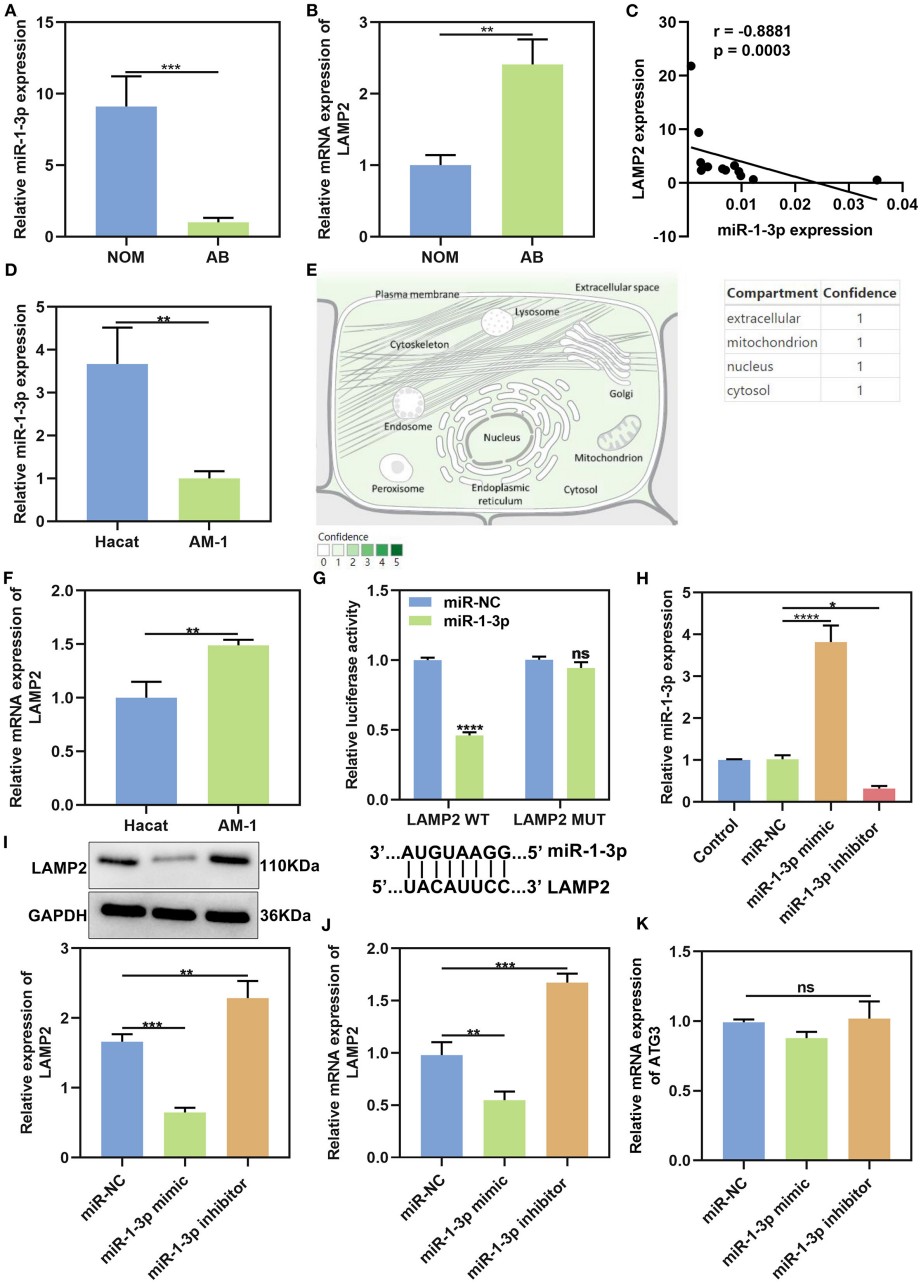
MiR-1-3p suppresses LAMP2 expression in AB. **(A,B)** qRT-PCR for the expression of miR-1-3p and LAMP2 in NOM and AB tissues. **(C)** Spearman correlation analysis of miR-1-3p expression and LAMP2 expression in AB tissues (*r* = −0.8881, *P* = 0.0003). **(D)** qRT-PCR for the expression of miR-1-3p in AM-1 cells and HaCat cells. **(E)** The distribution of miR-1-3p in cells. **(F)** qRT-PCR for the expression of LAMP2 in AM-1 cells and HaCat cells. **(G)** Assessment of relative luciferase activity in AM-1 cells after transfection with miR-1-3p mimic or miR-NC and schematic representation of the predicted miR-1-3p target sites within the 3'-UTR of LAMP2. **(H)** Assessment of the expression of miR-1-3p in AM-1 cells transfected with its mimic or inhibitor. **(I)** Western blot for LAMP2 expression in AM-1 cells transfected with miR-1-3p mimic or inhibitor. **(J)** qRT-PCR for detecting LAMP2 expression in AM-1 cells under transfection with miR-1-3p mimic or inhibitor. **(K)** qRT-PCR for examining ATG3 expression in AM-1 cells transfected with miR-1-3p mimic or inhibitor. *P < 0.05; **P < 0.01; ***P < 0.001; ****P < 0.0001; ns, not significant.

Previous studies have highlighted the roles of miR-1-3p in cancers. Bioinformatics analysis showed that miR-1-3p could bind to 3'UTR of LAMP2 mRNA among three autophagy-related proteins. We firstly analyzed the expression levels of miR-1-3p and LAMP2 in AB tissues by qRT-PCR. As a result, miR-1-3p was distinctly lowly expressed in AB than NOM tissues (*P* < 0.001; Figure 4A). On the contrary, LAMP2 mRNA in AB tissues displayed a higher level in comparison to NOM tissues (*P* < 0.01; Figure 4B). Spearman correlation analysis results suggested that miR-1-3p expression was negatively correlated with LAMP2 expression in AB tissues (*r* = −0.8881, *P* = 0.0003; Figure 4C). These data were indicative that LAMP2 might be a target mRNA of miR-1-3p in AB. MiR-1-3p expression was further detected in AM-1 and HaCat cells. Our results showed the down-regulation of miR-1-3p in AM-1 cells compared to HaCat cells (*P* < 0.01; Figure 4D). In Figure 4E, miR-1-3p was mainly expressed in extracellular, mitochondrion, nucleus, and cytosol. LAMP2 mRNA was significantly up-regulated in AM-1 cells compared to HaCat cells (*P* < 0.01; Figure 4F). MiRNAs mediate the silencing of target genes at the post-transcriptional level by binding to the 3'-untranslated region (3'-UTR) of mRNAs. By the miRanda database (http://www.miranda.org/), we found that LAMP2 was a potential target of miR-1-3p (Figure 4G). Luciferase assay confirmed that miR-1-3p may bind to LAMP2, and inhibited LAMP2 luciferase activity in AM-1 cells (*P* < 0.0001; Figure 4G). However, no inhibitory effect was found after mutation of this binding motif. To confirm whether miR-1-3p affected LAMP2 expression, miR-1-3p mimic or inhibitor was transfected into AM-1 cells. In Figure 4H, it was successfully overexpressed by miR-1-3p mimic transfection (*P* < 0.0001) while it was silenced after transfection with miR-1-3p inhibitor (*P* < 0.05). As expected, LAMP2 protein expression was decreased by miR-1-3p mimic (*P* < 0.001) and increased by its inhibitor (*P* < 0.01; Figure 4I). Similarly, miR-1-3p mimic significantly lowered the expression of LAMP2 mRNA in AM-1 cells (*P* < 0.01; Figure 4J). Meanwhile, LAMP2 expression was distinctly up-regulated when transfected by miR-1-3p inhibitor (*P* < 0.001). A previous study reported that ATG3 was a potential target of miR-1 in NSCLC cells (16). However, our data showed that miR-1-3p mimic or inhibitor did not significantly alter ATG3 expression in AM-1 cells (Figure 4K).

### miR-1-3p Restrains Proliferation and Facilitates Apoptosis in AB Cells

**Figure 5:**
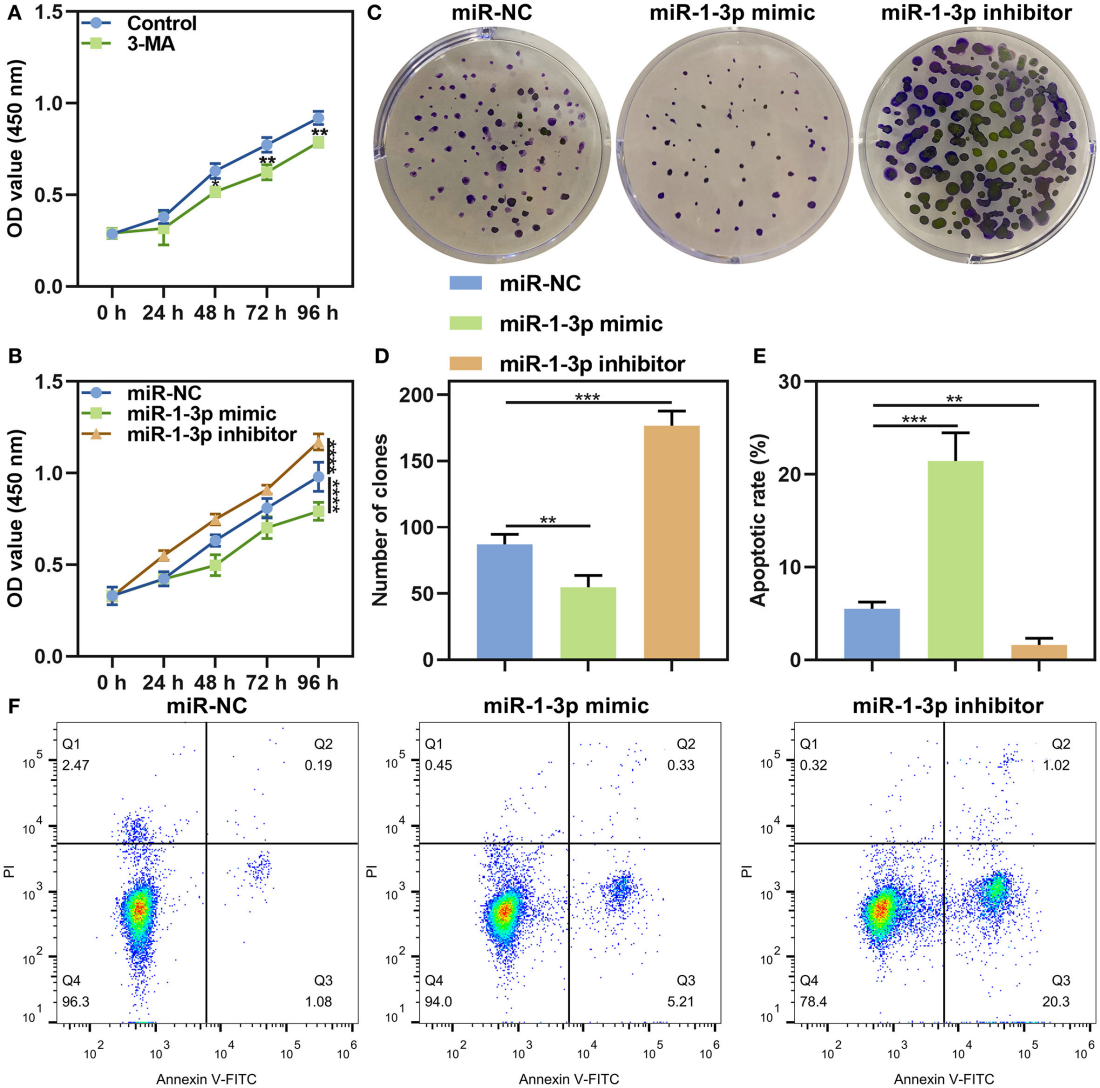
The roles of miR-1-3p on proliferation and apoptosis in AM-1 cells. **(A)** CCK-8 for cell viability of AM-1 cells treated with autophagy inhibitor 3-MA at 0, 24, 48, 72, and 96 h. **(B)** CCK-8 for cell viability of AM-1 cells transfected with miR-1-3p mimic or inhibitor at 0, 24, 48, 72, and 96 h. **(C,D)** The number of colonies of AM-1 cells with miR-1-3p mimic or inhibitor transfection. **(E,F)** Flow cytometry for detecting apoptosis of transfected AM-1 cells. *P < 0.05; **P < 0.01; ***P < 0.001; ****P < 0.0001.

Firstly, we observed the roles of autophagy on malignant phenotypes of AB. After AM-1 cells were treated with 3 mmol/L autophagy inhibitor 3-MA for 0, 24, 48, 72, and 96 h, cell viability was measured. As a result, 3-MA treatment distinctly suppressed cell proliferation (*p* < 0.05; Figure 5A), indicating that inhibiting autophagy could weaken malignant progression of AB. Then, the functions of miR-1-3p on AB progression were investigated in depth. As a result, its overexpression lowered the cell viability of AM-1 cells (*P* < 0.0001). Meanwhile, the cell viability of AM-1 cells was increased by its knockdown (*P* < 0.0001; Figure 5B). Clone formation ability was further evaluated. In Figures 5C,D, miR-1-3p mimic significantly decreased the number of clones (*P* < 0.01) and the opposite results were observed when transfected by miR-1-3p inhibitor (*P* < 0.001). Furthermore, AM-1 cells displayed elevated apoptotic levels following transfection by miR-1-3p mimic (*P* < 0.001; Figures 5E,F). However, its inhibitor decreased the apoptosis of AM-1 cells (*P* < 0.01). Collectively, miR-1-3p may restrain proliferation as well as facilitate apoptosis in AM-1 cells.

### miR-1-3p Reduces Migrated and Invasive Capacities in AB Cells

**Figure 6:**
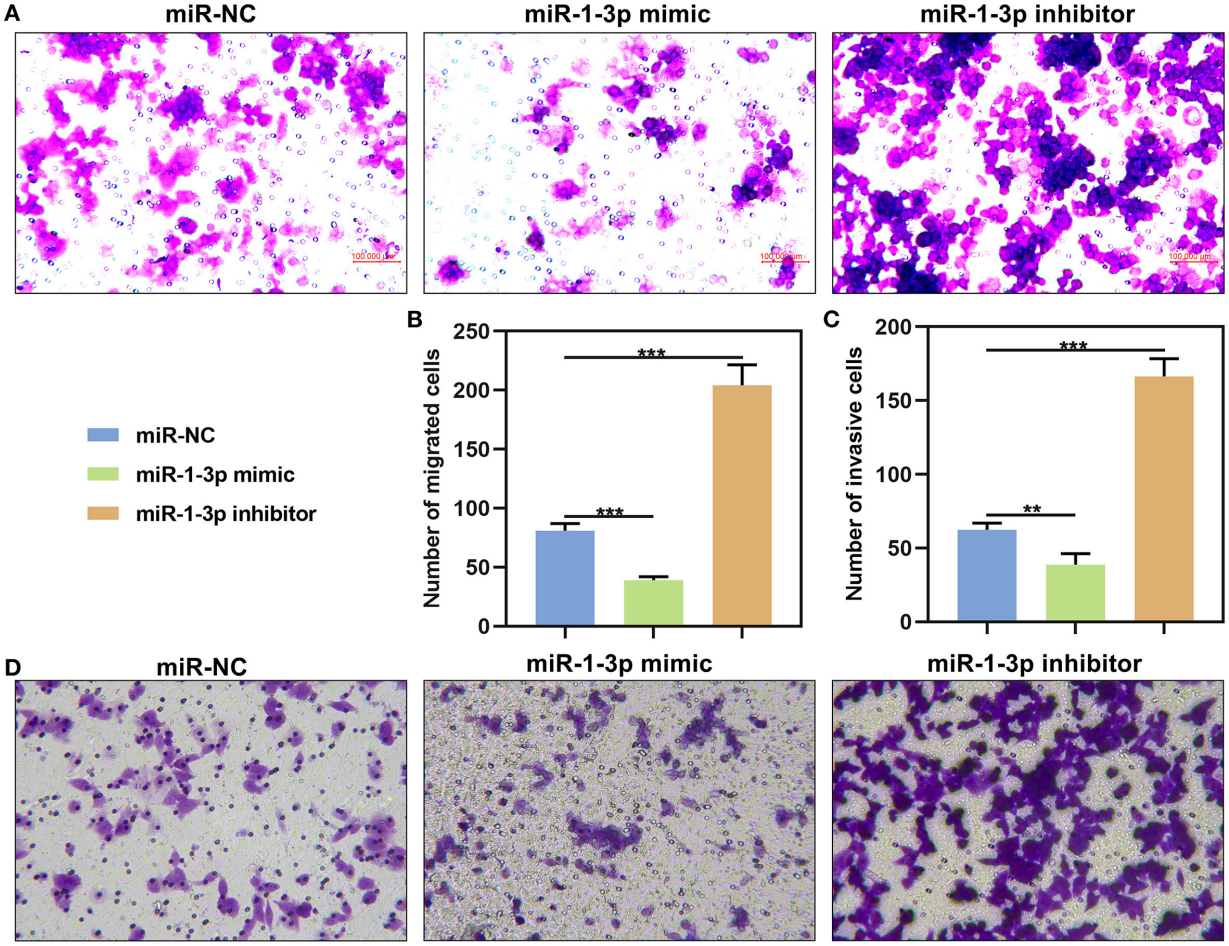
The effects of miR-1-3p on migration and invasion of in AM-1 cells. **(A,B)** Evaluation of the number of migrated AM-1 cells transfected with miR-1-3p mimic or inhibitor. **(C,D)** Detection of the number of invasive AM-1 cells after transfection. **P < 0.01; ***P < 0.001.

This study further evaluated migration and invasion of AB cells by transwell assays. As a result, the number of migrated AM-1 cells was markedly reduced by miR-1-3p mimic (*P* < 0.001), which was increased after transfection by its inhibitor (*P* < 0.001; Figures 6A,B). Furthermore, its knockdown restrained invasive capacities of AM-1 cells (*P* < 0.01) and the opposite consequences were investigated after overexpressing miR-1-3p (*P* < 0.001; Figures 6C,D).

### miR-1-3p Suppresses Malignant Phenotypes of AB Cells by Down-Regulating LAMP2

**Figure 7:**
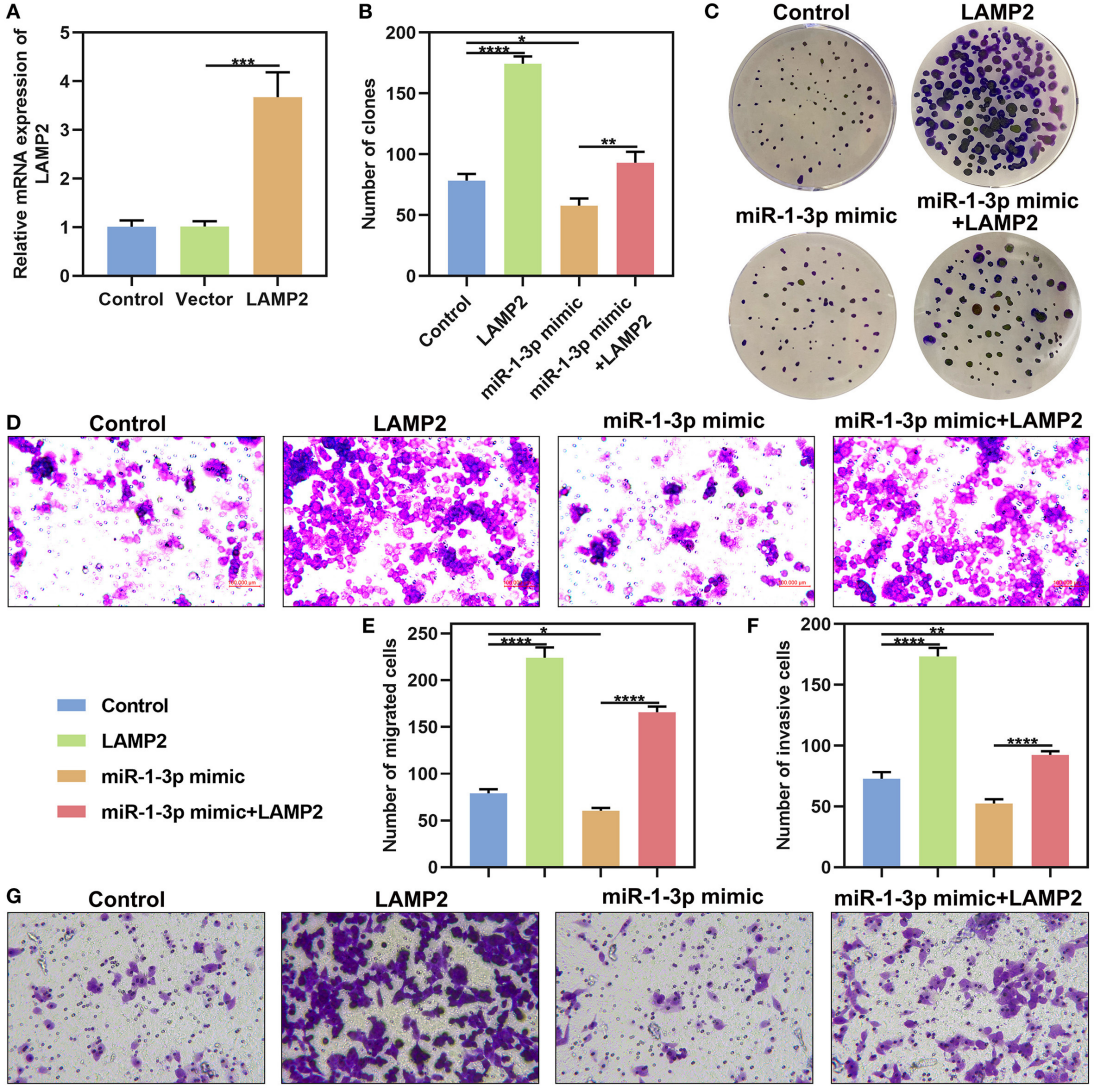
miR-1-3p suppresses colony formation, migration and invasion of AM-1 cells by down-regulating LAMP2. **(A)** qRT-PCR for evaluating the expression of LAMP2 in AM-1 cells with pcDNA3.1 LAMP2 plasmid transfection. **(B,C)** The number of colonies for AM-1 cells transfected with LAMP2 and/or miR-1-3p mimic. **(D–G)** The number of **(D,E)** migrated and **(F,G)** invasive AM-1 cells under transfection with LAMP2 and/or miR-1-3p mimic. *P < 0.05; **P < 0.01; ***P < 0.001; ****P < 0.0001.

LAMP2 was significantly overexpressed in AM-1 cells under transfection with pcDNA3.1 LAMP2 (*P* < 0.001; Figure 7A). The up-regulation significantly increased the number of clones of AM-1 cells (*P* < 0.0001; Figures 7B,C). Furthermore, the decrease in the number of clones induced by miR-1-3p overexpression was markedly ameliorated by LAMP2 upreg ulation (*P* < 0.01). We also found that LAMP2 overexpression distinctly enhanced migrated (*P* < 0.0001; Figures 7D,E) and invasive (*P* < 0.0001; Figures 7F,G) abilities of AM-1 cells. Meanwhile, its up-regulation ameliorated the reduction in migrated (*P* < 0.0001) and invasive (*P* < 0.0001) abilities of AM-1 cells induced by miR-1-3p mimic. Above findings were indicative that miR-1-3p suppressed malignant phenotypes of AB cells by down-regulating LAMP2.

## Discussion

Here, our research analyzed the dysregulated expression and clinical implications of autophagy-related proteins including Beclin1, LC3II/I, and LAMP2 in AB. Also, we found that LAMP2 might be a potential target mRNA of miR-1-3p in AB. This miRNA may suppress malignant phenotypes of AB cells by down-regulating LAMP2.

Studies have shown that epithelial cells of AB have stronger autophagy activity than mesenchymal cells, human odontogenic cells, and maxillary mesenchymal stem cells, indicating that autophagy may promote the cell viability of residual AB epithelial cells in the hypoxic tumor microenvironment that is in relation to local invasion of AB (7). Consistently, our data showed that inhibiting autophagy significantly weakened cell proliferation of AM-1 cells. BRAF mutation often occurs in AB and is associated with recurrence of AB (18, 19). It has been found that BRAF oncogene induces the expression of key autophagy markers including LC3 and Beclin1, suggesting that the recurrence of AB might be due to the activation of autophagy of residual AB cells after surgery (20). However, in this study, we found that there was no correlation between LC3 or Beclin1 expression and AB recurrence, which requires in-depth research.

Epithelial-mesenchymal transition (EMT) is a key process in the invasion as well as metastases of AB (21). Adherent epithelial cells into highly active mesenchymal cells are the early stage of tumor invasion and metastasis. For example, in liver cancer, starvation-induced autophagy promotes the expression of EMT-related molecular markers through the transforming growth factor β (TGF-β)/Smad3 signaling pathway, thereby enhancing the invasive ability of hepatoma cells (22). In bladder cancer, it has been also demonstrated that autophagy can activate TGF-β/Smad signaling pathway-mediated EMT to promote tumor cell invasion and metastasis (23). At present, it has been found that EMT-related transcription factors Slug, Snail and TGF-β are up-regulated and E-cadherin expression is down-regulated in AB tissues, suggesting EMT may be involved in the development of AB (24). In this study, western blot analysis results showed that in AB tissues, Beclin1 expression was down-regulated, while LC3 and LAMP2 were both up-regulated, indicating that autophagy was activated in AB. Combining previous studies, autophagy might increase the invasive ability of AB cells via promoting EMT process.

As shown in immunohistochemistry, the positive expression rate of LC3 and LAMP2 in the mandible of AB was significantly elevated compared to that in the maxilla. Because the incidence rate of AB in the mandible is significantly higher than that in the maxilla. Moreover, there is no report on the relationships between AB location and tumor biological behaviors. It is difficult to prove that the expression levels of LAMP2 and LC3 are in association with AB location (25). We speculate that in the mandible, blood supply is more singular, the bone is denser, and the tumor cells are more prone to hypoxia or energy deficiency compared to the maxilla, however, autophagy can supply energy for tumor cell proliferation. In this study, the cases of the AB occurring in the maxilla was small and the differences between the positive expression rates of LC3 and LAMP2 and AB location still need to be further validated in a larger cohort. In addition, the positive expression rate of LC3 in solid/polycystic AB was significantly higher than other classifications. It has been found that the recurrence rate of solid/polycystic AB is significantly higher than other classifications, suggesting that elevated levels of autophagy in solid/polycystic AB may be associated with tumor recurrence (26).

Currently, the molecular biology research on AB focuses on finding biomarkers. Combining with our previous human miRNA expression microarray results (six pairs of AB vs. NOM), among the down-regulated miRNAs, miR-1-3p in AB exhibited a down-regulated pattern than NOM tissues (27). Studies have demonstrated that it is down-regulated in a variety of tumors, which plays a role in inhibiting tumor cell proliferation as well as invasion (26, 28). Intriguingly, it can also influence the biological behavior of tumors by targeting and regulating autophagy-related molecules. In NSCLC, miRNA-1 binds to ATG3 to inhibit ATG3-mediated autophagy, thereby improving the cisplatin resistance of NSCLC cells (16). Through bioinformatics analysis, miR-1-3p might be an upstream regulatory molecule of LAMP2. The negative correlation between the expression of miR-1-3p and LAMP2 was found in AB. We found that there were miR-1-3p target sites within the 3′-UTR of LAMP2. Dual luciferase report confirmed that miR-1-3p could mediate LAMP2 expression by binding to the 3′-UTR of LAMP2. After validation, in AB cells, miR-1-3p up-regulation significantly suppressed LAMP2 expression at the mRNA and protein levels. Also, miR-1-3p restrained proliferation, migration and invasion of AB cells through down-regulating LAMP2. Several animal trials have reported the use of miR-1-3p as a target for cancer intervention or treatment. For instance, miR-1-3p may inhibit xenograft tumor growth of lung adenocarcinoma (29). MiR-1-3p up-regulation restrains hepatocellular carcinoma growth in mouse xenograft model (30). MiR-1-3p may overcome gefitinib resistance of lung cancer in tumor xenografts (31). In the formation and progression of tumors, lysosomes can increase the invasiveness of tumor cells by altering localization, volume, and composition, and releasing lysosomal enzymes (32, 33). LAMP2 mediates the fusion of autophagosomes and lysosomes during autophagy and plays an important role in autophagosome maturation (34). In colon cancer, LAMP2 is differentially expressed and has specific diagnostic value in the early stage of colon cancer as a molecular marker (35). In breast cancer, high expression of LAMP2 is also detected, and its high expression is significantly associated with tumor progression (36). In our research, LAMP2 was significantly up-regulated in AB tissues as well as epithelial-derived AM-1 cells, indicating that there could be an increase in autophagy activation in AB. LAMP2 up-regulation promoted colony formation, migrated and invasive abilities of AB cells. Our further study showed that LAMP2 was significantly negatively correlated with miR-1-3p in AB, suggesting that miR-1-3p may regulate autophagy by inhibiting LAMP2 expression at post-transcriptional levels in AB, thereby participating in AB progression.

## Conclusion

In our study, we examined the abnormal expression of autophagy-related proteins including LAMP2, Beclin1, and LC3, suggesting the activation of autophagy in AB. Further study showed that LAMP2 might be a potential target mRNA of miR-1-3p in AB. MiR-1-3p silenced LAMP2 expression by binding to the 3'-UTR of LAMP2, thereby suppressing malignant phenotypes of AB. Thus, our research may provide a novel insight into the mechanisms of AB.

## Data Availability Statement

The original contributions presented in the study are included in the article/supplementary material, further inquiries can be directed to the corresponding author/s.

## Ethics Statement

The studies involving human participants were reviewed and approved by the Ethics Committee of School and Hospital of Stomatology, China Medical University (2016-12). The patients/participants provided their written informed consent to participate in this study.

## Author Contributions

MZ conceived and designed the study. XN and BH conducted most of the experiments and data analysis, and wrote the manuscript. XQ, JL, and LC participated in collecting data and helped to draft the manuscript. All authors contributed to the article and approved the submitted version.

## Conflict of Interest

The authors declare that the research was conducted in the absence of any commercial or financial relationships that could be construed as a potential conflict of interest.

## Abbreviations

AB: ameloblastoma
NOM: normal oral mucosa
LAMP2: lysosomal associated membrane protein 2
Beclin1: B-cell lymphoma-2-interacting protein-1
LC3: microtubule-associated protein 1 light chain 3
miRNA: microRNA
qRT-PCR: quantitative Real-time PCR
CCK-8: cell counting kit-8
EMT: epithelial-mesenchymal transition.
